# Neurovascular-immune-clearance remodeling in brain aging

**DOI:** 10.3389/fimmu.2026.1950144

**Published:** 2026-10-29

**Authors:** Yuxin Liu, Ronghao Yang, Haocai Chang

**Affiliations:** 1 MOE Key Laboratory of Laser Life Science & Institute of Laser Life Science, College of Biophotonics, School of Optoelectronic Science and Engineering, South China Normal University, Guangzhou, China; 2 Guangdong Provincial Key Laboratory of Laser Life Science, College of Biophotonics, School of Optoelectronic Science and Engineering, South China Normal University, Guangzhou, China

**Keywords:** blood brain barrier, brain aging, cellular senescence, glymphatic system, meningeal lymphatics, neuroinflammation, neurovascular unit

## Abstract

Brain aging involves concurrent changes in cerebral vessels, immune regulation, and waste clearance, yet these processes are often studied separately. This fragmented approach limits understanding of how dysfunction in one system influences the others and contributes to age-related neurological disorders. This review summarizes current evidence on the neurovascular-immune-clearance axis, focusing on neurovascular unit deterioration, impaired glymphatic and meningeal lymphatic transport, neuroimmune remodeling, and the cell-specific mechanisms that connect these processes. We further compare their shared and disease-dependent roles in Alzheimer’s disease, vascular cognitive impairment, and late-life depression. Available imaging, fluid, proteomic, and epigenetic biomarkers are evaluated for their ability to measure different components of this axis, and emerging interventions are organized according to their predominant effects on vascular support, clearance function, immune signaling, and cellular senescence. Current evidence indicates that brain aging is characterized by interacting and partially compensable functional bottlenecks rather than a single linear pathological pathway. Integrating these mechanisms provides a framework for interpreting heterogeneous aging phenotypes, selecting complementary biomarkers, and developing mechanism-guided interventions. Future progress will depend on longitudinal human validation and direct demonstration that target engagement produces durable neurological benefit.

## 1 Introduction

Brain aging cannot be reduced to the accumulation of independent molecular lesions. Rather, it involves coordinated and cell-type-specific remodeling across vascular, immune, and clearance compartments (1). Here, we organize current evidence around three interacting modules: the neurovascular unit (NVU), the CNS border parenchymal immune interface, and the glymphatic meningeal lymphatic clearance network.

Within the NVU, aging-associated endothelial dysfunction and senescence-like changes, deficient pericyte remodeling, and cerebrovascular network rarefaction compromise blood brain barrier (BBB) integrity, capillary flow distribution, and tissue oxygenation (2–4). The immune interface comprises parenchymal microglia, border-associated macrophages located in the meninges, choroid plexus, and perivascular spaces, and adaptive immune cells residing at the CNS borders (5, 6). Notably, parenchymal border macrophages regulate cerebrospinal fluid (CSF) flow dynamics, whereas age-associated meningeal T-cell activation can impair lymphatic drainage through interferon-γ signaling (6, 7).

The clearance network couples perivascular CSF influx and interstitial fluid (ISF) efflux to downstream meningeal lymphatic drainage, thereby facilitating the removal of metabolic waste products and aggregation-prone proteins. Recent work further identifies arterial pulsatility and slow vasomotion as important contributors to this transport process (8, 9). During aging, these modules become functionally interdependent: alterations in vascular function and perivascular macrophage homeostasis can disrupt CSF transport, whereas age related immune signaling and extracellular matrix remodeling in the meninges impair lymphatic drainage and promote waste retention and immune dysfunction (6, 7, 10).

Waste retention may amplify inflammatory stress, while age related neuroinflammation, including microglial activation and complement pathway engagement, may further compromise vascular and lymphatic homeostasis, establishing a plausible self-reinforcing feedback loop (11, 12). Importantly, selected components of this axis remain experimentally modifiable in aged mice: exposure to a young systemic environment restores cortical capillary density and BBB integrity, whereas systemic platelet factor 4 enhances hippocampal neurogenesis and cognitive function (13, 14). This review therefore adopts the neurovascular immune clearance triad as a conceptual framework for examining how coupled dysfunction across these systems contributes to brain aging and for identifying measurable and potentially tractable points of intervention (15) (**Figure 1**).

**Figure 1:**
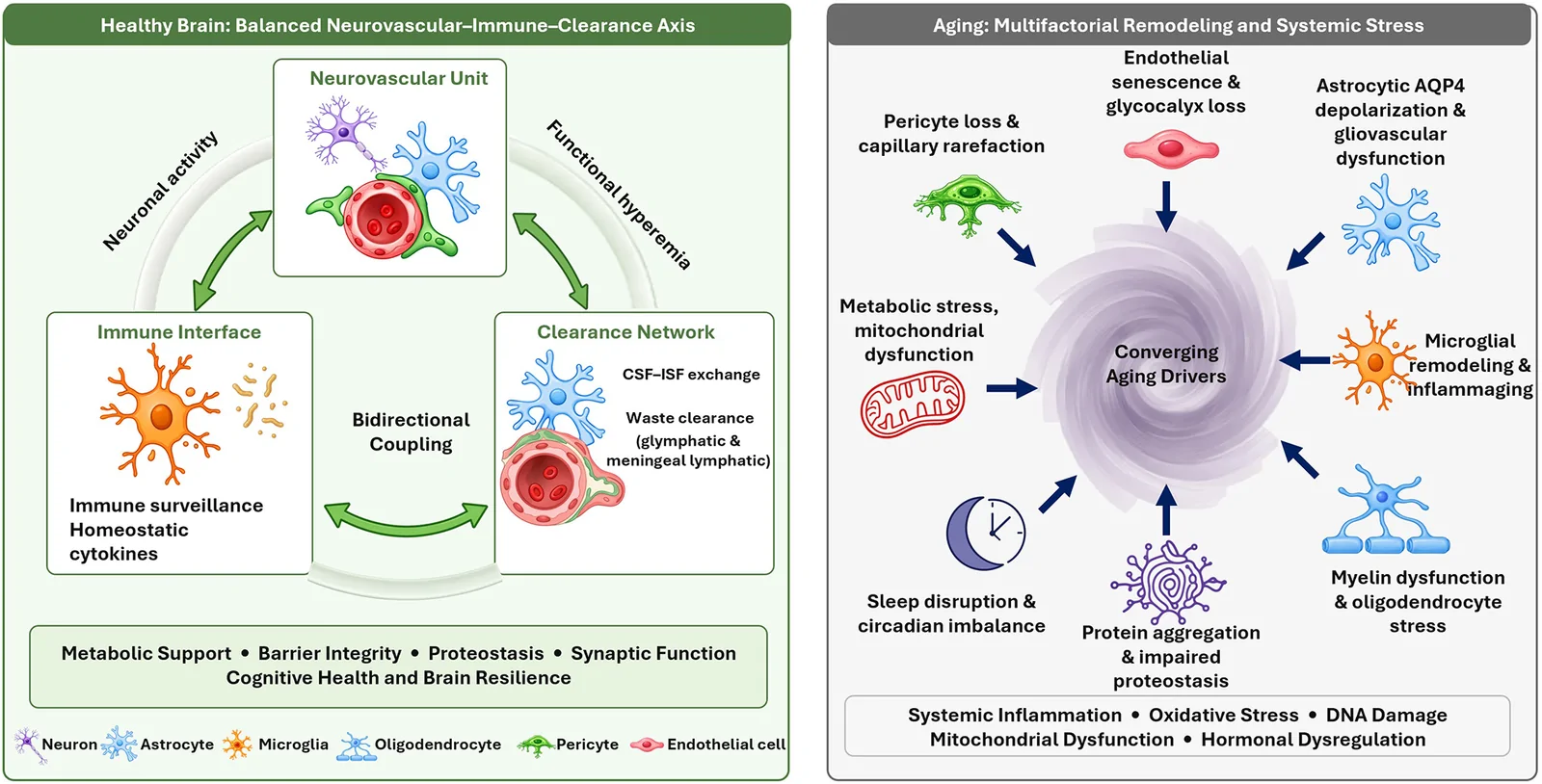
Integrated neurovascular-immune-clearance axis during brain aging. In the healthy brain, the neurovascular unit, immune interfaces, and clearance pathways operate as a coordinated system that supports metabolic homeostasis, BBB integrity, proteostasis, synaptic function, and cognitive resilience. Bidirectional communication among neuronal activity, vascular responses, immune surveillance, CSF-ISF exchange, and glymphatic-meningeal lymphatic drainage maintains tissue stability. During aging, endothelial senescence and glycocalyx loss, pericyte depletion, capillary rarefaction, astrocytic aquaporin-4 (AQP4) depolarization, microglial remodeling, oligodendrocyte and myelin dysfunction, impaired proteostasis, mitochondrial stress, and sleep or circadian disruption converge with systemic inflammation, oxidative stress, DNA damage, and hormonal dysregulation. When compensatory capacity is exceeded, these changes promote neurovascular failure, immune dysregulation, and impaired fluid and solute clearance. Dysfunction in each compartment can increase stress on the others, creating a self-reinforcing cycle of barrier disruption, chronic inflammation, defective phagocytosis, reduced meningeal lymphatic drainage, and waste accumulation. Arrows indicate functional interactions rather than a fixed linear sequence.

## 2 Neurovascular unit aging drives barrier breakdown and clearance failure

The NVU coordinates two functions that are central to brain homeostasis: selective exchange across the BBB and the adjustment of local blood flow to neuronal demand. These functions depend on interactions among endothelial cells, pericytes, vascular smooth muscle cells, basement membrane components, astrocytic endfeet, and neurons (16). Aging weakens both barrier control and vascular responsiveness. This dual failure provides a mechanistic link between NVU decline and the immune and clearance abnormalities discussed in subsequent sections.

Brain endothelial cells are positioned at the blood brain interface and therefore form the first point of failure (**Figure 1**). Senescence-like endothelial changes increase inflammatory secretory activity, weaken barrier integrity, induce adhesion molecules, and facilitate peripheral immune cell entry into the brain (3, 17). The luminal endothelial glycocalyx is also altered with age. In aged mice, this layer becomes thinner and loses mucin-type O-glycosylation; experimental disruption of these glycans increases vascular leakage, whereas their restoration improves BBB function and reduces neuroinflammatory changes (18). Endothelial aging should therefore be viewed not simply as a loss of tightness, but as a transition from a selective barrier toward a more inflammatory vascular interface.

Pericyte dysfunction affects a different aspect of NVU control. Pericytes contribute to microvascular homeostasis, blood brain barrier regulation, and the matching of capillary flow to local metabolic demand. Experimental pericyte degeneration disrupts neurovascular coupling and limits oxygen delivery, while broader evidence links pericyte dysfunction to age related small vessel injury and impaired vascular stability (19–21). Pericyte aging is therefore better understood as a loss of microvascular control that can aggravate barrier and metabolic stress.

Astrocytic endfeet add a further layer of vascular stability. They maintain extensive contact with the cerebrovascular surface and support blood brain barrier regulation, local blood flow, nutrient handling, and perivascular solute transport (22). Aging and disease related astroglial atrophy may reduce these homeostatic and protective functions, thereby weakening coordination among neuronal activity, vascular tone, and brain fluid transport (23, 24).

The vascular basement membrane and associated extracellular matrix are also remodeled across the lifespan. Human brain microvessel proteomics identifies age-dependent changes in collagen, perlecan, integrin, and transporter expression, although major tight junction proteins do not appear to decline uniformly with normal aging (25). These findings support matrix remodeling as part of NVU aging, but they do not establish that basement membrane thickening alone blocks ISF movement. The matrix supports communication among endothelial cells, pericytes, and astrocytes. Age related changes in its composition may reduce the ability of the vasculature to maintain homeostasis and respond to injury.

These changes link NVU aging to dysfunction across the interconnected vascular, immune, and clearance systems. Barrier leakage increases the exposure of perivascular and parenchymal cells to plasma proteins, adhesion signals, and circulating leukocytes, thereby favouring immune remodeling (26, 27). In parallel, arterial pulsatility and slow vasomotion contribute to cerebrospinal-fluid influx and glymphatic transport (8, 9). Loss of vascular flexibility and capillary coordination may therefore impair not only oxygen delivery but also the physical conditions required for efficient solute clearance. NVU aging is thus an upstream loss of vascular control: barrier failure alters the immune environment, whereas disturbed vascular dynamics constrain perfusion and brain fluid clearance.

## 3 Impaired glymphatic transport and meningeal lymphatic drainage in aging

The brain removes extracellular proteins, soluble metabolites, and excess fluid through several overlapping routes. Perivascular transport moves CSF into contact with ISF, while meningeal and extracranial lymphatic vessels carry part of the CSF burden towards cervical lymph nodes. These routes interact, but they do not form a single uninterrupted channel, because CSF can leave the cranial compartment through several anatomically distinct pathways with different capacities for compensation (28–31).

### 3.1 Glymphatic transport and changes in CSF and ISF exchange

Glymphatic transport depends partly on the perivascular distribution of AQP4 in astrocytic endfeet. Loss of this spatial organisation reduces CSF tracer influx and slows the removal of interstitial tracers, even when total AQP4 expression does not fall to the same extent. Aging also alters perivascular macrophages, which regulate arterial motion and CSF flow. AQP4 redistribution and macrophage dysfunction should therefore be treated as related contributors to impaired transport rather than as separate or sufficient causes (32–34).

Vascular motion and sleep state also shape glymphatic transport. During nonrapid eye movement sleep, slow changes in norepinephrine, cerebral blood volume, and CSF movement occur in a coordinated pattern (9, 35, 36). Experimental manipulation of these vascular oscillations changes CSF entry in mice, which supports a causal role for slow vasomotion (9, 36). Increased arterial pulsation and stronger AQP4 polarisation also accompany enhanced amyloid clearance after multisensory stimulation, although this result comes from transgenic Alzheimer’s disease (AD) models rather than normal aging (35–39). Human measurements of brain parenchymal resistance further associate deeper sleep and greater delta activity with conditions that favour glymphatic exchange, but these measurements remain indirect (40, 41).

Changes in glymphatic transport can affect extracellular protein handling. AQP4 deletion reduces the movement of extracellular tau from brain tissue into CSF and then towards deep cervical lymph nodes. In P301S mice, this disruption increases phosphorylated tau deposition and neurodegeneration. Loss of perivascular AQP4 localisation also increases amyloid beta burden, while reduced AQP4 function weakens the amyloid clearing effect of sensory stimulation (42–46). These studies support a role for glymphatic transport in proteostasis, but they do not show that glymphatic impairment alone initiates human neurodegenerative disease.

### 3.2 Meningeal and cervical lymphatic drainage

Meningeal lymphatic vessels provide an anatomical route between the cranial compartment and extracranial lymphatic networks (47). Dural vessels near venous sinuses carry CSF components and immune material, while lymphatic structures near the skull base and nasopharynx direct fluid towards cervical lymph nodes (48). Deep and superficial cervical routes contribute differently to CSF outflow, and obstruction of one route can recruit partial compensation through another (49–52). The drainage system is therefore better described as a branched network than as a single basal exit pathway.

Aging alters both upstream lymphatic structures and downstream vessel function (53). The nasopharyngeal lymphatic plexus shows structural regression in aged mice, whereas cervical collecting vessels develop weaker contraction and poorer valve coordination. Improving cervical vessel contraction increases CSF drainage, which indicates that part of the functional decline remains reversible (49, 54). More recent work also shows that mechanical stimulation of superficial cervical lymphatics can increase CSF outflow in aged mice, further supporting the importance of extracranial pumping mechanisms (54, 55).

The aging meningeal environment imposes additional restrictions on lymphatic drainage. T cells accumulate near dural sinuses and increase interferon gamma signalling, which reduces drainage towards deep cervical lymph nodes. Neutralising this signal improves lymphatic function without producing a comparable increase in glymphatic influx, suggesting that immune aging can act directly at the outflow stage (6, 56). At the same time, collagen accumulates around meningeal lymphatic vessels. The resulting change in matrix organisation and stiffness disrupts lymphatic junctions and limits lymphangiogenesis, linking immune signalling, fibroblast activity, and drainage failure within the dura (6, 10, 57).

Human evidence supports the presence of related structures but remains less mechanistically complete. Magnetic resonance imaging can visualise signals consistent with dural lymphatic channels and connections to cervical lymph nodes, while physiological monitoring links sleep with changes in brain tissue resistance that are compatible with altered fluid exchange (40, 58, 59). These techniques do not yet measure every step of CSF transport or lymphatic pumping directly. Results from aged mice should therefore inform, rather than define, the interpretation of human brain clearance.

### 3.3 Functional links between glymphatic transport and lymphatic drainage

Glymphatic transport and lymphatic drainage share sequential steps because some solutes move from the interstitial compartment into CSF before reaching cervical lymph nodes (60). Extracellular tau transport provides direct experimental support for this sequence. However, CSF also uses nasal, dural, cervical, perineural, and vascular routes, and the contribution of each pathway changes with pressure, anatomy, and tracer properties (31, 42, 49). The two systems therefore contain serial elements, but they do not operate as an obligatory linear pipeline.

The pathological effects of reduced drainage depend on the degree of compensation and the disease model. Sustained manipulation of dorsal meningeal lymphatic vessels reduces macromolecular drainage but does not necessarily increase total amyloid plaque burden, which indicates that other clearance mechanisms can limit the effect of one impaired route (31, 61). By contrast, impaired AQP4 dependent transport increases extracellular tau retention, and reduced cervical lymphatic pumping slows CSF outflow (42, 54). Aging therefore creates several potential bottlenecks rather than one uniform clearance failure.

Taken together, current evidence supports a restrained model. Within the brain, changes in AQP4 localisation, vascular motion, perivascular macrophages, and sleep physiology may reduce CSF and ISF exchange. At the meningeal and cervical boundaries, lymphatic regression, weaker vessel contraction, inflammatory signalling, and matrix remodelling may restrict outflow. Dysfunction at either level can increase the load placed on the remaining pathways, but protein accumulation and neuroimmune activation also depend on compensatory drainage, protein production, disease stage, and vascular function (6, 10, 61).

## 4 Neuroimmune remodeling and inflammaging

The concept of absolute immune privilege no longer adequately describes the aging brain. The blood brain barrier limits unrestricted leukocyte entry, but the meninges, choroid plexus, perivascular spaces, and dural sinuses form active immune interfaces (62). Dural lymphatic vessels transport fluid and immune material toward cervical lymph nodes, while T cells survey antigens near the dural sinuses. Meningeal B cells and border associated macrophages occupy distinct niches and contribute to local surveillance rather than simply representing cells that have escaped from the circulation (7, 63, 64). Aging changes these interfaces, but it does not abolish them. Increased interferon gamma signaling in the aged meninges impairs lymphatic drainage, whereas human imaging suggests changes in dural lymphatic channels and cervical lymph nodes without directly measuring every step of lymphatic function (6, 65).

Microglia undergo heterogeneous changes rather than switching uniformly from a resting to an activated state (66). Single cell and spatial analyses identify reductions in homeostatic programs alongside the emergence of inflammatory, interferon responsive, lipid processing, and phagocytic states. These changes differ across brain regions, with white matter showing particularly strong inflammatory and glial responses (67, 68). Metabolic dysfunction contributes to this remodeling. In aged mice, excessive prostaglandin E2 (PGE2) signaling through prostaglandin E2 receptor EP2 (EP2) limits glucose use in myeloid cells, whereas EP2 inhibition improves bioenergetics, reduces inflammatory activity, and restores aspects of memory performance (69). This result supports metabolic plasticity in aged myeloid cells, but it does not establish EP2 inhibition as a clinically available treatment. It is also more accurate to treat age associated and disease associated microglial states as overlapping programs than as two completely separate cell identities (70).

Astrocyte aging is similarly diverse. Normal aging produces spatially restricted reactive programs rather than a uniform conversion into a single harmful subtype. Inflammatory transcripts increase in selected astrocyte populations, while genes involved in metabolic support, ion regulation, and cellular maintenance decline in a region dependent manner (67, 71). Aging also reduces the ability of neighboring astrocytes to restore vascular endfoot coverage after local injury, which may weaken recovery of the gliovascular interface (72). Inflammatory astrocyte signaling can impair barrier properties, but this effect depends on the stimulus and experimental system; tumor necrosis factor induced astrocyte reactivity, for example, disrupts barrier function in a human cell based model through STAT3 and SERPINA3 signaling (73). These findings support a spectrum of astrocyte states and argue against retaining the binary A1 and A2 classification as the main framework for aging (74).

Adaptive immune cells become more visible within aged brain tissue, particularly in white matter. Cytotoxic CD8 positive T cells accumulate in the aging mouse central nervous system and contribute to axonal injury and functional decline. Their effects depend partly on local glial signals: interferon responsive oligodendrocytes and microglia cluster near these T cells, while CXCL10 produced within aged white matter promotes recruitment and retention through CXCR3 (75–77). This pathway provides a direct link between innate glial remodeling and adaptive immune entry. It should not, however, be generalized to all aging brain regions or all T cell subsets. Some resident T cell populations may exert regulatory or protective functions, and most causal evidence for age related axonal injury comes from mice (78). Human studies in neurodegenerative disease support brain infiltration by distinct CD8 positive populations, but disease associated infiltration cannot be assumed to reproduce normal aging exactly (79).

Complement signaling adds another layer to glial remodeling. Complement proteins can mark synaptic structures and alter their uptake by microglia or astrocytes, but the outcome depends on the disease model, cell state, and synapse type. In a combined amyloid and tau model, astrocytes remove excitatory and inhibitory synapses through a C1q dependent mechanism and can partly compensate when microglial uptake is impaired (80). Complement activity is also restrained by protective mechanisms. TREM2 can bind C1q and limit complement mediated synapse loss during neurodegeneration, showing that microglial signaling does not simply amplify complement activity in one direction (81). Complement is therefore better viewed as a context dependent regulator of synaptic remodeling than as an invariably destructive cascade (82). Broad inhibition may preserve synapses in selected pathological settings, but selective targeting is preferable because the same pathway participates in debris recognition and tissue maintenance.

Inflammaging emerges from sustained interactions among altered immune cells, vascular dysfunction, extracellular proteins, and senescent cell populations (83). Senescence cannot be identified by a single marker, and expression of p16, p21, or inflammatory factors does not by itself establish an irreversible senescent state. Nevertheless, p16 positive myeloid populations accumulate in aged mouse brain and express senescence associated and inflammatory programs that can promote peripheral immune cell chemotaxis. Genetic clearance of these cells partly restores the immune landscape and preserves cognition, although the intervention removes senescent cells in both central and peripheral tissues (84). Whole body senescent cell clearance also reduces brain inflammation and improves memory in aged mice, again leaving the relative contribution of brain and systemic senescence unresolved (85).

The senescence associated secretory phenotype provides a plausible mechanism through which a relatively small number of cells can affect neighboring tissue. Senescent cells can release cytokines, chemokines, growth factors, and matrix modifying enzymes, but the composition of this secretome varies with cell type and trigger (86). Human brain organoids accumulate senescent cells during prolonged culture, and intermittent dasatinib plus quercetin treatment reduces inflammatory and senescence related signatures in this model (87). These data support the biological tractability of senescence, not established clinical efficacy. The SToMP-AD trial was designed primarily to assess safety, feasibility, and central nervous system exposure to dasatinib and quercetin rather than cognitive benefit (88).

Neuroimmune aging therefore reflects interacting changes in brain borders, resident glia, infiltrating lymphocytes, complement signaling, and cellular senescence. No single component explains the entire process. Microglial metabolism, astrocyte reactivity, T cell recruitment, and SASP signaling remain experimentally modifiable, but most intervention evidence comes from mice or model systems. Future studies should separate normal aging from neurodegenerative disease, distinguish central from systemic treatment effects, and validate biomarkers that reflect defined mechanisms rather than nonspecific inflammation.

## 5 Cellular effectors of integrated neurovascular, immune, and clearance dysfunction

Age related brain dysfunction emerges from reciprocal changes among neurons, glial cells, and vascular cells rather than from the isolated failure of one cell population. These changes weaken the matching of neuronal demand to vascular supply, reduce the maintenance of perivascular interfaces, increase the burden placed on cellular degradation systems, and limit microvascular repair (**Figure 2**).

**Figure 2:**
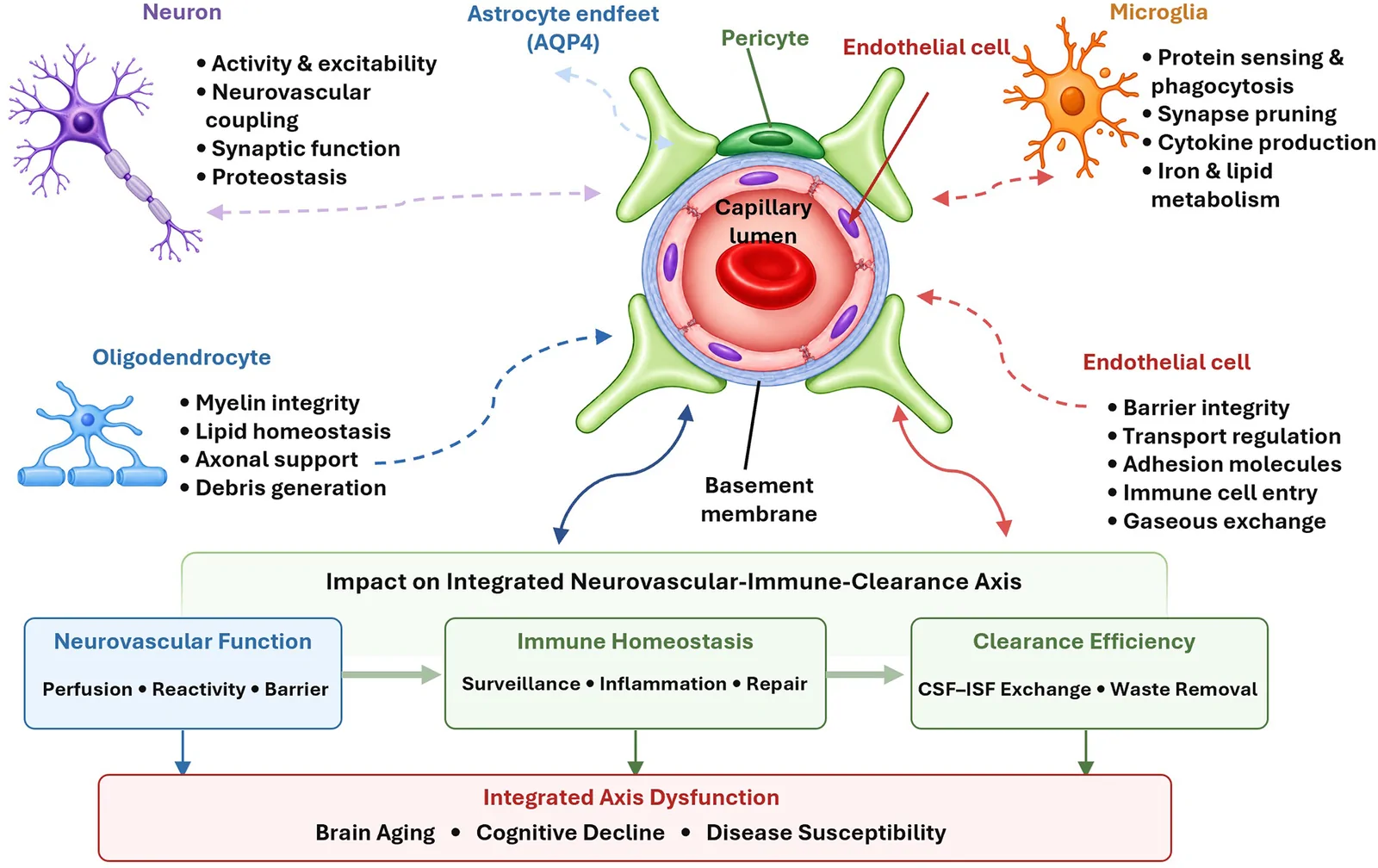
Cellular effectors remodel the neurovascular-immune-clearance axis. Neurons, astrocytes, microglia, oligodendrocytes, pericytes, and endothelial cells jointly maintain the structure and function of the neurovascular unit. Neuronal activity supports neurovascular coupling, synaptic signaling, and proteostasis; astrocytic endfeet regulate aquaporin-4 polarity, perivascular organization, CSF-ISF exchange, and ionic and water homeostasis; microglia contribute to protein sensing, phagocytosis, synaptic remodeling, cytokine production, and iron and lipid metabolism; and oligodendrocytes preserve myelin integrity, axonal support, and lipid homeostasis. Pericytes, endothelial cells, and the basement membrane coordinate capillary tone, barrier integrity, molecular transport, immune cell entry, and vascular homeostasis. Age-related dysfunction in these cellular compartments compromises neurovascular function, including perfusion, vascular reactivity, and barrier regulation; disrupts immune homeostasis through altered surveillance, inflammatory responses, and repair processes; and reduces clearance efficiency by impairing CSF-ISF exchange and waste removal. Their combined deterioration promotes disruption of the integrated neurovascular-immune-clearance axis, thereby contributing to brain aging, cognitive decline, and increased susceptibility to neurological disease. Arrows indicate functional interactions among cellular components and downstream system-level consequences rather than a fixed linear sequence.

Neuronal activity and vascular function form a bidirectional relationship. Older adults show weaker frontal neurovascular responses during cognitively demanding tasks, and this reduction is associated with poorer cognitive performance. However, neurovascular coupling is not uniformly lost with age. Evoked hyperemia remains largely preserved in some mice aged 18 months despite reduced baseline and activity related oxygen availability, indicating that age effects depend on the brain region, task, and experimental model (4, 89). Vascular deterioration can also suppress neuronal function. Longitudinal imaging shows that microvessel regression reduces nearby neuronal activity through changes in mitochondrial metabolism and glutamate production (90). Neuronal hypoactivity and vascular decline should therefore be viewed as mutually reinforcing processes rather than as a one way loss of neuronal control over blood flow.

Astrocytes maintain the interface between neural tissue and cerebral vessels. Perivascular localization of AQP4 supports exchange between CSF and ISF, whereas loss of this localization reduces tracer exchange and amyloid β (Aβ) clearance in mice (91). Astrocytes also respond to local cell loss by extending processes that restore vascular coverage. This replacement response is delayed in aged mice, suggesting that aging weakens repair of the gliovascular interface rather than simply causing uniform endfoot withdrawal (72). Astrocyte aging may therefore impair both perivascular organization and recovery after local injury, although neither process alone explains the full decline in brain clearance.

Neuronal proteostasis and microglial processing become increasingly coupled with age. Neuronal proteins turn over more slowly in aged mice, and many proteins with reduced degradation also aggregate or accumulate within microglia. Synaptic proteins are strongly represented among this transferred material, indicating that microglia participate in the disposal of proteins released from, secreted by, or engulfed from neurons (92). At the same time, microglia undergo progressive transcriptional and epigenetic changes involving lipid handling, iron metabolism, cytokine production, and immune activation. These trajectories differ by sex and do not support a single uniform aged phenotype (93). Aging therefore increases the material presented to microglia while also changing their capacity to process it. This interpretation is more defensible than describing aged microglia as universally inactive phagocytes or exclusively inflammatory cells.

Microglia and oligodendrocytes jointly maintain myelin integrity. Adult microglia regulate myelin growth and limit structural degeneration, partly through signaling between microglial TGFβ1 and oligodendrocyte TGFβR1. Loss of microglia produces abnormal myelin growth, degeneration, and an oligodendrocyte state marked by altered lipid metabolism (94). Age related changes in microglia may therefore weaken myelin maintenance and increase the amount of lipid rich material requiring cellular processing. Current evidence supports an increased metabolic and degradative burden, but it does not establish that myelin fragments mechanically obstruct glymphatic flow. The latter claim should be omitted unless direct fluid dynamic evidence becomes available (92, 94).

Mural cell aging limits the ability of the microcirculation to maintain vascular tone and repair local damage. Focal pericyte loss causes abnormal capillary dilation, heterogeneous red blood cell distribution, flow steal, stalling, and eventual vessel regression. Neighboring pericytes can restore endothelial coverage, but this remodeling proceeds more slowly in aged mice. Importantly, focal pericyte loss does not invariably produce immediate BBB leakage (2). Aging also reduces the responsiveness of precapillary sphincters and first order capillaries, with loss of mural cell processes and weaker control of pressure and flow (95). Pericyte and precapillary mural cell dysfunction should therefore be linked primarily to impaired capillary regulation and repair, with barrier failure treated as a possible but context dependent consequence.

Brain endothelial cells add a distinct form of cellular vulnerability. The luminal glycocalyx becomes thinner and loses specific mucin type O-glycosylation patterns with age. Experimental restoration of these glycans improves barrier integrity and reduces inflammatory and cognitive abnormalities in aged mice, supporting a functional role for endothelial surface remodeling rather than a simple decline in tight junction abundance (18). Endothelial DNA repair deficiency also produces senescence associated signaling, increased adhesion molecule expression, barrier leakage, and peripheral immune cell infiltration, although this accelerated model cannot be equated directly with natural aging (3). Endothelial aging is therefore best described as a shift in barrier regulation, transport, adhesion, and inflammatory signaling rather than as uniform endothelial loss.

These cellular processes converge without progressing at the same rate or in the same direction. Neurons and vessels influence each other’s metabolic function; astrocytes lose part of their capacity to preserve perivascular organization; microglia face increasing neuronal and myelin derived material while undergoing intrinsic state changes; oligodendrocyte lipid regulation becomes dependent on altered glial support; and vascular cells show reduced control and repair. Their interaction can amplify neurovascular, immune, and clearance dysfunction, but the extent of this amplification varies across brain regions, sexes, disease states, and stages of aging (4, 18, 93).

## 6 Shared neurovascular–immune–clearance dysfunction across brain disorders

The preceding sections described vascular, immune, and clearance dysfunction at the mechanistic level. At the disease level, these processes often coexist, but they do not form a single obligatory pathway. Their relative importance varies with the initiating pathology, disease stage, and capacity of alternative transport and repair mechanisms.

### 6.1 Shared dysfunction across age related brain disorders

In AD, barrier dysregulation, impaired extracellular protein handling, and altered glial responses frequently occur together (96, 97). Loss of perivascular AQP4 localization reduces CSF and ISF exchange and increases Aβ deposition in mice, while enhancement of glymphatic transport can promote amyloid removal in transgenic models (8, 91). Meningeal lymphatic dysfunction also alters microglial responses and reduces the efficacy of antiamyloid immunotherapy in mice (98). These findings support a modifying role for clearance pathways, but they do not establish lymphatic failure as a necessary cause of amyloid deposition (99). Sustained manipulation of dorsal meningeal lymphatic vessels changes macromolecular drainage without necessarily changing total amyloid plaque burden, indicating that other clearance routes can compensate (61).

Parkinson disease (PD) provides related but more limited evidence (100). Dynamic contrast enhanced magnetic resonance imaging shows reduced meningeal lymphatic flow and delayed deep cervical lymph node perfusion in patients with idiopathic PD. In α-synuclein mouse models, obstruction of lymphatic flow increases α-synuclein pathology and worsens motor and memory deficits (101). These observations support an association between impaired drainage and disease progression, but they do not show that drainage dysfunction initiates sporadic PD. Human imaging measures flow indirectly, and the temporal sequence remains difficult to determine (65).

In vascular cognitive impairment and dementia, cerebrovascular disease provides the dominant point of entry, but its downstream effects are heterogeneous. Large and small vessel disease, ischemic injury, hemorrhage, hypoperfusion, and reduced vascular transport can all contribute to cognitive impairment. BBB leakage and water exchange also differ across cerebral small vessel disease subtypes, arguing against a uniform pattern of vascular failure (102, 103). Vascular injury may alter ISF balance and promote inflammatory responses, but the contribution of clearance dysfunction probably varies with lesion type, burden, and anatomical distribution (104, 105).

Perioperative neurocognitive disorders illustrate how an acute systemic challenge can expose preexisting brain vulnerability. Prospective human data associate postoperative delirium with perioperative changes in blood brain barrier permeability, CSF IL-6, and markers of neuronal stress (106). Increased CSF fibrinogen after surgery is also associated with barrier permeability and selected inflammatory and neuronal injury markers, although it does not independently predict delirium severity in a small cohort (107). These findings support dynamic interactions among vascular permeability, inflammation, and neuronal injury (108). They do not yet justify describing human glymphatic transport as temporarily paralyzed after surgery.

Late life depression is more heterogeneous than the original formulation implied. Vascular disease, inflammation, sensory loss, metabolic dysfunction, and psychosocial stress can contribute to different clinical subgroups rather than defining one common mechanism (109). Peripheral inflammation is associated with changes in tissue water and functional connectivity within depression related networks, but available cross sectional data do not establish causality (110). Epigenetic age is associated bidirectionally with late life brain health events at the population level, although it cannot identify whether vascular, immune, or clearance dysfunction is responsible in an individual patient (111). Direct impairment of brain waste clearance in late life depression remains insufficiently established and should not be presented as a defining feature.

Across these disorders, neurovascular, immune, and clearance abnormalities are best viewed as shared modifiers of susceptibility and progression. Protein aggregation is more prominent in AD and PD, vascular injury dominates many forms of vascular cognitive impairment, systemic inflammation is especially relevant after surgery, and late life depression contains several biological subtypes. The systems overlap, but their contribution is neither equal nor inevitable.

### 6.2 Multimodal assessment of system dysfunction

Structural magnetic resonance imaging based brain age models provide global measures of biological vulnerability rather than direct measures of a particular mechanism. Newer approaches estimate the pace of aging from a single brain image and associate faster scores with poorer physical and cognitive function (112). Brain clocks derived from magnetic resonance imaging and electroencephalography also show substantial variation across populations and are influenced by socioeconomic and health related disparities (113). Brain age should therefore be interpreted as an integrated risk marker, not as a specific measure of vascular, immune, or clearance dysfunction.

More targeted imaging can characterize individual components. Dynamic contrast enhanced magnetic resonance imaging measures aspects of blood brain barrier permeability and meningeal lymphatic flow, while high resolution magnetic resonance imaging can visualize dural lymphatic channels and their connections with cervical lymph nodes (65, 101, 114). Diffusion based measures of perivascular transport remain indirect and are sensitive to white matter architecture, vascular disease, and acquisition methods. The term “AQP4 MRI” should therefore be avoided unless a technique measures AQP4 specifically. Current human studies more often combine imaging proxies of perivascular transport with CSF or tissue measurements of AQP4.

Fluid biomarkers provide complementary but mechanistically distinct information. Plasma phosphorylated tau 217 identifies amyloid and tau pathology with accuracy similar to established CSF measures, but it should be described as an AD pathology biomarker rather than a direct measure of clearance efficiency (115). Plasma proteomic models can estimate organ specific aging and associate accelerated brain or immune aging with adverse health outcomes (116, 117). Paired CSF and plasma proteomics further identify changes in protein concentration ratios associated with aging and cognitive impairment, offering information about compartmental transport, inflammation, and barrier regulation (118). No single protein measurement captures all three systems.

Epigenetic clocks estimate systemic biological aging from DNA methylation patterns. Accelerated epigenetic age is associated with stroke, dementia, and late life depression at the population level, and brain health events may also accelerate epigenetic aging (111). These clocks provide information about accumulated biological stress but do not localize dysfunction to the neurovasculature, immune system, or brain clearance pathways. They should therefore complement, rather than replace, imaging and fluid biomarkers.

A multimodal framework should combine measurements that provide nonredundant information: structural brain aging, vascular permeability and reactivity, perivascular or lymphatic transport, disease specific protein pathology, inflammatory activity, and systemic biological age. A composite dysfunction index is a reasonable research objective, but it is not yet a validated clinical tool. Such an index would require longitudinal validation, external replication, standardized acquisition, and evidence that it predicts outcomes more accurately than its individual components (**Figure 3**).

**Figure 3:**
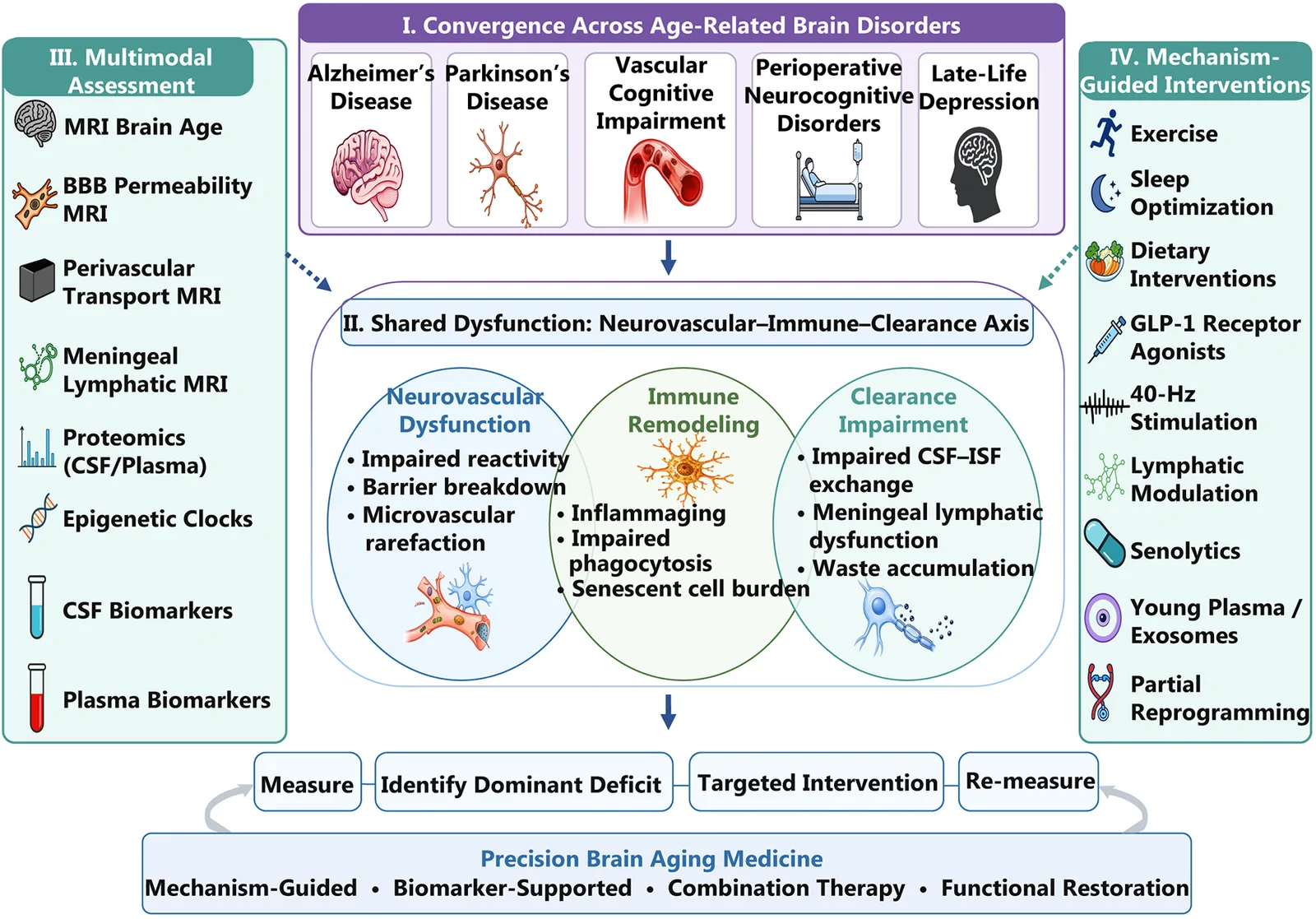
Convergence of diseases, biomarkers and therapeutic interventions. The image presents a conceptual framework linking age-related brain disorders with shared dysfunction of the neurovascular-immune-clearance axis, multimodal biomarker assessment, and mechanism-guided therapeutic interventions. Alzheimer’s disease, Parkinson’s disease, vascular cognitive impairment, perioperative neurocognitive disorders, and late-life depression may converge on overlapping neurovascular, immune, and clearance abnormalities despite their distinct clinical and pathological features. These abnormalities include impaired vascular reactivity, blood–brain barrier disruption, and microvascular rarefaction; inflammaging, defective phagocytosis, and increased senescent-cell burden; and disrupted CSF-ISF exchange, meningeal lymphatic dysfunction, and waste accumulation. Complementary biomarkers include MRI-based brain-age estimates, BBB permeability imaging, perivascular transport and meningeal lymphatic MRI, CSF and plasma proteomics, epigenetic clocks, and fluid biomarkers. Candidate interventions comprise exercise, sleep and dietary optimization, GLP-1 receptor agonists, 40-Hz stimulation, lymphatic modulation, senolytics, young plasma-derived extracellular vesicles, and partial reprogramming. The lower workflow illustrates a precision brain-aging strategy in which repeated assessment is used to identify the dominant functional deficit, guide intervention selection, and evaluate target engagement and treatment response.

## 7 Therapeutic targeting of the neurovascular-immune-clearance axis

Interventions for brain aging can be organized according to the function they primarily modify: vascular and metabolic support, brain fluid clearance, or immune and senescence related signaling. This classification is useful for interpreting mechanism, but it should not imply strict target specificity. Exercise, sleep, metabolic drugs, lymphatic manipulation, and senescence directed therapies often affect more than one system. Their evidence also ranges from human randomized trials to early clinical feasibility studies and experimental work in mice (**Figure 3**).

### 7.1 Restoring vascular and metabolic support

Vascular function remains modifiable in later life. Aerobic exercise can increase cerebral blood flow and reduce cerebrovascular resistance and arterial stiffness in older adults, although the extent to which these vascular changes account for cognitive improvement remains uncertain (119). Multidomain programs that combine physical activity, nutrition, cognitive engagement, and vascular risk management produce modest cognitive benefits and may be more appropriate for heterogeneous aging populations than a single lifestyle intervention (120).

Metabolic therapies may provide additional support in selected disease settings. Lixisenatide was associated with slower motor deterioration over 12 months in early Parkinson disease, but the trial did not establish whether the effect arose from direct neuronal signaling, systemic metabolic improvement, vascular protection, or immune modulation (121). GLP-1 receptor agonists should therefore be presented as disease focused candidates with possible relevance to brain aging, not as established rejuvenation agents.

Repair of the vascular interface offers a more direct experimental strategy. Restoration of selected endothelial glycocalyx glycans improves barrier integrity and reduces inflammatory abnormalities in aged mice, indicating that endothelial surface regulation remains therapeutically responsive (18). Translation will require approaches that preserve vascular specificity and avoid altering glycosylation in unrelated tissues.

### 7.2 Enhancing physiological clearance

Sleep supports brain fluid transport through coordinated changes in neural activity, vascular volume, and CSF movement. Slow vasomotion during nonrapid eye movement sleep contributes to fluid influx in mice, while emerging human methods can monitor physiological changes associated with solute exchange (9, 122). These findings justify sleep optimization as part of healthy aging, but they do not establish treatment of sleep disruption as a direct method for reversing protein accumulation.

Clearance can also be modified experimentally through sensory, lymphatic, and vascular interventions. Multisensory stimulation at 40 Hz increases CSF influx, ISF efflux, arterial pulsatility, perivascular AQP4 localization, and amyloid removal in transgenic mice (8). Pharmacological enhancement of cervical lymphatic contraction and mechanical stimulation of superficial cervical lymphatics increase CSF drainage in aged mice (54, 55). Enhancement of meningeal lymphatic function can also modify microglial responses and improve the activity of antiamyloid antibodies in experimental Alzheimer disease (98).

These studies demonstrate that clearance pathways are experimentally tractable. They do not yet establish clinical efficacy, because human lymphatic anatomy, treatment delivery, long term safety, and appropriate physiological endpoints remain incompletely defined.

### 7.3 Modulating immune aging and cellular senescence

Immune aging may be modified by targeting cell metabolism, inflammatory signaling, or senescent cell survival. Inhibition of PGE2 signaling through the EP2 receptor restores aspects of myeloid cell metabolism and cognitive function in aged mice, suggesting that inflammatory metabolic states retain some reversibility (69). Neutralization of IFN-γ improves meningeal lymphatic drainage in aged mice without producing a comparable increase in glymphatic influx, indicating that immune signaling can act selectively at the lymphatic outflow stage (6).

Senolytic treatment remains at an earlier translational stage (123). A small phase 1 study of dasatinib plus quercetin established limited central nervous system exposure and short-term feasibility in mild Alzheimer disease, but it did not demonstrate cognitive or neuroimaging benefit (124). The relevant senescent cell populations, the relative contributions of central and peripheral effects, and the consequences of repeated cell depletion remain unresolved.

Young plasma derived extracellular vesicles and partial reprogramming represent broader attempts to modify cellular aging programs. Young plasma vesicles improve mitochondrial metabolism and functional performance in aged mice, while pulsed expression of reprogramming factors restores selected progenitor populations and molecular features of the aged neurogenic niche (125, 126). Both approaches remain preclinical. Their translation will require identification of active components, tissue specific delivery, and safeguards against unwanted growth or loss of cellular identity.

The principal targets, experimental models, measured effects, and evidence stages of the interventions discussed above are summarized in **Table 1**.

**Table 1:** Representative experimental and clinical interventions targeting neurovascular, immune, and clearance dysfunction in brain aging.

Intervention	Primary target	Experimental or clinical model	Main effects and proposed mechanism	Reference
Progressive aerobic exercise training	Cerebrovascular resistance, carotid arterial stiffness and global cerebral blood flow	Randomized trial of 73 cognitively normal older adults, mean age approximately 68 years; one year of moderate to vigorous aerobic exercise versus stretching and toning	Increased global cerebral blood flow and reduced carotid stiffness and cerebrovascular resistance. Improvements in vascular measures were associated with better memory, but mediation was not established.	(119)
Lixisenatide, a GLP-1 receptor agonist	GLP-1 receptor signaling; metabolic and neuronal stress pathways	Phase 2 randomized trial of 156 adults aged 40-75 years with Parkinson disease diagnosed within three years and receiving stable dopaminergic treatment; 12 months of lixisenatide or placebo	Reduced progression of motor disability relative to placebo. The trial did not directly demonstrate restoration of vascular, immune or clearance functions, and gastrointestinal adverse effects were common.	(121)
Multisensory 40 Hz stimulation	Cortical VIP interneurons, arterial pulsatility, astrocytic AQP4 localization and meningeal lymphatic vessels	5×FAD mice aged 6 months exposed to one hour of combined auditory and visual 40 Hz stimulation; control frequencies included 8 and 80 Hz	Increased CSF influx and ISF efflux, arterial pulsatility, perivascular AQP4 localization and meningeal lymphatic vessel diameter. Inhibition of VIP interneurons or AQP4 attenuated amyloid clearance.	(8)
VEGF-C mediated lymphatic enhancement combined with antiamyloid antibodies	VEGFR3 signaling in meningeal lymphatic endothelial cells	5×FAD mice with established amyloid pathology treated with VEGF-C based lymphatic manipulation and antiamyloid monoclonal antibodies	Enhanced meningeal lymphatic function increased antibody associated Aβ removal and modified microglial responses. The results support improved treatment delivery and clearance in mice rather than direct reversal of AD.	(98)
Topical prostaglandin F_2_α	Contractile smooth muscle cells of cervical collecting lymphatic vessels	C57BL/6 mice aged 18 or 22 months, compared with two month old controls; topical application over cervical lymphatic vessels	Aging reduced lymphatic contraction and forward pumping. Prostaglandin F_2_α restored slowed lymphatic flow and increased CSF drainage from the cranial compartment.	(54)
Transdermal mechanical stimulation of superficial cervical lymphatics	Smooth muscle covered superficial cervical lymphatic vessels and their valves	Prox1-GFP mice aged 80-95 weeks, compared with adults aged 8-12 weeks; regulated mechanical compression through intact facial and neck skin	Repeated compression increased CSF outflow through superficial cervical lymphatics and largely corrected the drainage deficit in aged mice. It had little effect on spontaneous lymphatic contractions.	(55)
Pharmacological or genetic inhibition of myeloid EP2	PGE2 signaling through EP2 in microglia and peripheral myeloid cells	Naturally aged mice, mainly 20-23 months old; pharmacological EP2 inhibition and myeloid specific genetic manipulation	Restored glucose utilization and mitochondrial bioenergetics in myeloid cells, reduced inflammatory activity and improved hippocampal plasticity and memory. Central and peripheral myeloid effects were not completely separable.	(69)
IFN-γ neutralization	IFN-γ signaling in meningeal lymphatic endothelial cells, including IFNGR1, IFNGR2 and lymphatic junction regulation	Mice aged 20-24 months; 200 µg anti-IFN-γ intraperitoneally every 72 hours for eight weeks	Increased drainage of CSF delivered ovalbumin to deep cervical lymph nodes. The treatment did not increase meningeal lymphatic coverage or glymphatic influx, indicating an effect mainly on lymphatic outflow.	(6)
Dasatinib plus quercetin senolytic treatment	Senescent cell survival pathways and the senescence associated secretory phenotype; exact CNS cell target not established	Open label phase 1 study involving five participants with early symptomatic AD; mean age 76 ± 5 years; intermittent treatment over 12 weeks	Dasatinib entered the CSF in four participants, whereas quercetin was not consistently detected. The study supported feasibility and short term safety but found no significant cognitive or neuroimaging improvement.	(124)
Small extracellular vesicles from young plasma	PGC-1α dependent mitochondrial biogenesis and energy metabolism; vesicular miRNA cargo	Male mice aged 21 months received 200 µl young plasma sEVs from two-month-old male donors seven times over two weeks; separate long-term cohorts began treatment at 20 months	Increased hippocampal PGC-1α, ATP synthesis, mitochondrial respiration and mitochondrial content. Treatment improved memory and endurance, but the responsible cargo probably extends beyond the identified miRNAs.	(125)
Pulsed partial reprogramming with OSKM	Neural stem cells, progenitor cells and neuroblasts in the subventricular zone	Inducible OSKM mice aged 18-20 or 24-26 months; whole body or subventricular zone targeted pulsed reprogramming	Restored neuroblast and neural progenitor proportions toward younger levels, improved selected transcriptomic aging signatures and increased production of new neurons. Cognitive benefit and long-term human safety were not established.	(126)
Restoration of brain endothelial glycocalyx glycosylation	Brain endothelial C1GALT1 and B3GNT3; core 1 mucin type O-glycosylation	mice aged 17 months received endothelial targeted AAV-C1GALT1, AAV-B3GNT3 or control AAV; barrier assessment after eight weeks and behavioral testing after twelve weeks	Both constructs reduced BBB leakage. B3GNT3 overexpression also improved Y maze and contextual fear performance and reduced microglial and astrocytic activation related signatures. C1GALT1 did not produce the same behavioral benefit.	(18)

As summarized in **Table 1**, most interventions affect more than one component of brain aging. Exercise, lymphatic manipulation, metabolic drugs, and senolytics may influence vascular, immune, clearance, and systemic pathways simultaneously, which complicates attribution to a single mechanism.

The strength of evidence also varies. Lifestyle and selected metabolic interventions have entered randomized human trials, whereas lymphatic manipulation, circulating vesicles, senolytics, and partial reprogramming remain largely preclinical or limited to early feasibility studies. Improvements in isolated biomarkers or behavioral outcomes should therefore not be interpreted as integrated restoration of brain function.

Combination strategies should be guided by the dominant functional deficit and evidence of target engagement rather than by empirical addition of treatments. Sequential intervention may improve mechanistic interpretation and limit toxicity. Timing is also important: vascular risk reduction may be most effective before substantial structural damage, clearance enhancement may be more useful while extracellular proteins remain accessible, and senescence directed or reprogramming approaches may require narrow exposure windows to avoid excessive cell loss or loss of cellular identity.

## 8 Future perspectives and concluding remarks

Further progress requires tools that connect intervention, target engagement, and functional outcome within the same individual. Imaging approaches should quantify regional vascular reactivity, barrier permeability, and brain fluid transport rather than relying only on structural atrophy or global brain age. Molecular measurements should distinguish protein pathology, immune activation, cellular senescence, and systemic aging instead of combining them into a nonspecific inflammatory signal.

A multimodal dysfunction index could integrate vascular, clearance, immune, and molecular measurements. However, such an index should remain a research objective until it undergoes longitudinal validation, external replication, and standardization across imaging and laboratory platforms. It must also show that changes in the composite score correspond to durable improvements in cognition, mobility, or disease progression rather than only to short term changes in biomarkers.

The evidence summarized in **Table 1** supports functional plasticity across several mechanisms involved in brain aging. It does not yet support whole brain rejuvenation as a clinical outcome. The most realistic near term goal is to identify the dominant and measurable dysfunction in each individual, restore that function safely, and determine whether the intervention reduces stress on connected systems. Moving from broad antiaging claims toward interventions guided by mechanism and biomarkers will be essential for translating experimental findings into clinically meaningful brain health.
